# Genome-Wide Analysis of DNA Methylation, Copy Number Variation, and Gene Expression in Monozygotic Twins Discordant for Primary Biliary Cirrhosis

**DOI:** 10.3389/fimmu.2014.00128

**Published:** 2014-03-28

**Authors:** Carlo Selmi, Francesca Cavaciocchi, Ana Lleo, Cristina Cheroni, Raffaele De Francesco, Simone A. Lombardi, Maria De Santis, Francesca Meda, Maria Gabriella Raimondo, Chiara Crotti, Marco Folci, Luca Zammataro, Marlyn J. Mayo, Nancy Bach, Shinji Shimoda, Stuart C. Gordon, Monica Miozzo, Pietro Invernizzi, Mauro Podda, Rossana Scavelli, Michelle R. Martin, Michael F. Seldin, Janine M. LaSalle, M. Eric Gershwin

**Affiliations:** ^1^Division of Rheumatology and Clinical Immunology, Humanitas Clinical and Research Center, Milan, Italy; ^2^Division of Rheumatology, Allergy, and Clinical Immunology, University of California at Davis, Davis, CA, USA; ^3^BIOMETRA Department, University of Milan, Milan, Italy; ^4^Liver Unit and Center for Autoimmune Liver Diseases, Humanitas Clinical and Research Center, Milan, Italy; ^5^National Institute of Molecular Genetics (INGM), Milan, Italy; ^6^University of Texas Southwestern, Dallas, TX, USA; ^7^Mt. Sinai University, New York, NY, USA; ^8^Clinical Research Center, National Nagasaki Medical Center, Nagasaki, Japan; ^9^Henry Ford Hospital, Detroit, MI, USA; ^10^Department of Pathophysiology and Transplantation, University of Milan, Milan, Italy; ^11^Division of Pathology, Fondazione IRCCS Cà Granda Ospedale Maggiore Policlinico, Milan, Italy; ^12^Genome Center and M.I.N.D. Institute, University of California at Davis, Davis, CA, USA; ^13^Department of Biochemistry and Molecular Medicine, University of California at Davis, Davis, CA, USA; ^14^Department of Internal Medicine, University of California at Davis, Davis, CA, USA

**Keywords:** autoimmune cholangitis, epigenetics, environment

## Abstract

Primary biliary cirrhosis (PBC) is an uncommon autoimmune disease with a homogeneous clinical phenotype that reflects incomplete disease concordance in monozygotic (MZ) twins. We have taken advantage of a unique collection consisting of genomic DNA and mRNA from peripheral blood cells of female MZ twins (*n* = 3 sets) and sisters of similar age (*n* = 8 pairs) discordant for disease. We performed a genome-wide study to investigate differences in (i) DNA methylation (using a custom tiled four-plex array containing tiled 50-mers 19,084 randomly chosen methylation sites), (ii) copy number variation (CNV) (with a chip including markers derived from the 1000 Genomes Project, all three HapMap phases, and recently published studies), and/or (iii) gene expression (by whole-genome expression arrays). Based on the results obtained from these three approaches we utilized quantitative PCR to compare the expression of candidate genes. Importantly, our data support consistent differences in discordant twins and siblings for the (i) methylation profiles of 60 gene regions, (ii) CNV of 10 genes, and (iii) the expression of 2 interferon-dependent genes. Quantitative PCR analysis showed that 17 of these genes are differentially expressed in discordant sibling pairs. In conclusion, we report that MZ twins and sisters discordant for PBC manifest particular epigenetic differences and highlight the value of the epigenetic study of twins.

## Introduction

Primary biliary cirrhosis (PBC) is a female-predominant autoimmune liver disease affecting the small interlobular bile ducts, ultimately leading to periportal fibrosis and cirrhosis (1). Similar to most autoimmune diseases, PBC onset results from the interplay of genomic predisposition and environmental factors (2–5) with a possible role for sex factors (6). Recent genome-wide association studies (GWAS) have reported consistent associations with polymorphisms of genes such as *IL12RA* and *HLA class II* in subgroups of patients with PBC (7–13) and a pathway analysis was recently performed (13). PBC concordance rates in dizygotic (DZ) and monozygotic (MZ) twins are significantly different being 0 and 63%, respectively, thus supporting the role of both genetic and environmental factors (14) with the latter supported also by epidemiology (15, 16).

Promoter methylation influences gene expression (GEX) and our group recently reported differences in the DNA methylation and expression of two X-linked genes (*PIN4* and *CLIC2*) in MZ twins discordant for PBC (17). On the other hand, copy number variations (CNV) are the result of duplications and other rearrangements (18) occur *de novo* at much higher rates than single nucleotide variants, and may regulate GEX (19). While sharing their genomic sequence, MZ twins may develop different phenotypes over the years because of increasing differences in DNA methylation (20) and CNV (21, 22).

We have taken advantage of a unique DNA collection of identical and non-identical twins with PBC and performed a genome-wide investigation to determine differences in DNA methylation, CNV, and GEX. Our data identify 17 candidate genes that are significantly under- or up-regulated in affected individuals and we suggest that these might constitute new candidates as disease markers of genetic determinants. The value of this approach is highlighted and suggests the need for the study of a large number of patients and cell subpopulations (23) to support this thesis.

## Materials and Methods

### Subjects

Blood samples from three MZ twins pairs discordant for PBC whose zygosity had been determined using microsatellite analysis (Ballestar) and eight sister pairs of similar age (within 5 years) discordant for PBC studied (Table 1). Serum antimitochondrial antibodies (AMA) were positive at indirect immunofluorescence in all patients with PBC and none of the healthy twins and sisters. In these subjects, PBC was excluded when serum AMA was negative and serum alkaline phosphatase was within normal range on two different occasions. Genomic DNA was isolated from peripheral blood mononuclear cells (PBMCs) using the QIAamp Blood Midi Kit (Qiagen, Valencia, CA, USA) and stored at −20°C until used. Additional blood samples were obtained using Tempus™ Blood RNA Tubes (Applied Biosystems, Foster City, CA, USA) that were stored at −20°C until mRNA was extracted using the RNeasy Mini Kit (Qiagen, Valencia, CA, USA) and then stored at −80°C. This study was performed in compliance with the ethical standards of medicine and, following approval by the local IRB, informed consents were obtained from all patients and controls in accordance with the Declaration of Helsinki.

**Table 1:** **Summary of the patients with PBC and the corresponding healthy sibling and twin sisters utilized in the study**.

PBC case #	Age (years)	Serum AMA	Control # (twin/sibling)	Age	Serum AMA
2	60	Pos	1 (MZ twin)	60	Neg
9	60	Pos	52 (MZ twin)	60	Neg
24	64	Pos	57 (MZ twin)	64	Neg
4	62	Pos	10 (Sister)	59	Neg
5	55	Pos	14 (Sister)	59	Neg
6	52	Pos	11 (Sister)	55	Neg
12	61	Pos	7 (Sister)	64	Neg
13	70	Pos	8 (Sister)	68	Neg
15	54	Pos	16 (Sister)	57	Neg
27	45	Pos	26 (Sister)	43	Neg
34	41	Pos	33 (Sister)	45	Neg
35	64	Pos	50 (Sister)	60	Neg

### Methylated DNA immunoprecipitation and methylation analysis

DNA samples of three MZ twin sets (#1/2, 9/52, and 24/57; see Table 1) were sonicated and then immunoprecipitated with a monoclonal antibody that specifically recognizes 5-methylcytidine (Roche NimbleGen, Madison, WI, USA). DNA fragments were converted into PCR-amplifiable OmniPlex™ Library molecules flanked by universal primer sites and the library amplified by PCR using universal primers and a limited number of cycles. Immunoprecipitated and reference DNA were tagged, respectively, with cyanine-5 (Cy5) and cyanine-3 (Cy3)-labeled random 9-mers and then hybridized by the NimbleGen Array Hybridization Kit (Roche NimbleGen, Madison, WI, USA).

A four-plex array was custom-designed to include 998 X chromosome and 18,086 randomly selected autosomal chromosome promoter sites (Roche NimbleGen, Madison, WI, USA) and samples analyzed following the manufacturers protocols. First, Nimblescan software (Roche NimbleGen, Madison, WI, USA) was utilized for DNA methylation data analysis using a threshold *p*-value of 0.05 equivalent to 1.31 based on the Gaussian distribution of data. Second, exclusive elements corresponding to specific microarray probes were identified in affected and healthy subjects and peaks found only in either group were selected for further analysis. Third, elements of interest were inserted into the UCSC Genome Browser (GRCh36/hg19) to identify corresponding genes.

### Copy number variation analysis

Copy number variation analysis was performed on genomic DNA from one MZ twin set (#1/2; see Table 1) using the Infinium R HD Assay Super platform (Illumina, San Diego, CA, USA): in particular, we utilized the HumanOmni1-Quad BeadChip that includes markers derived from the 1000 Genomes Project, all three HapMap phases, and recently published studies (7, 9, 24, 25) as well as adequate tools for quality control, CNV calling, and validation. The protocol included the initial DNA preamplification, fragmentation, and precipitation. Data obtained from four-plex chips were analyzed using iScan and Illumina BeadArray system (Illumina, San Diego, CA, USA) followed by the GenomeStudio software (Illumina, San Diego, CA, USA). The position of each probe and the number of copies for each probe were determined using the PennCNV platform based on a hidden Markov model algorithm (26). The UCSC Genome Browser was then used to determine the genes involved and the number of CNV.

### Microarray gene expression analysis

We utilized RNA samples from eight pairs of sisters of similar age (Table 1) discordant for PBC. In the first part, we performed a whole-genome microarray comparison of transcripts to detect consistently up- or down-regulated genes in affected subjects. We obtained biotin-labeled cRNA using the Illumina R TotalPrep RNA Amplification Kit (Illumina, San Diego, CA, USA) and used the whole-genome Gene Expression Direct Hybridization Assay (Illumina, San Diego, CA, USA) including 24,500 transcripts. Microarrays were scanned using the BeadArray Reader (Illumina Inc., San Diego, CA, USA) and data were processed using BeadStudio software (Illumina Inc.). Expression data were quantified using a cut-off for significant gene differences of *p* < 0.05 with a twofold difference in expression as described elsewhere (27).

### RT-PCR expression analysis

Real-time PCR was utilized to analyze samples prepared from 1 μg total RNA according to high-capacity cDNA reverse transcription kit (Applied Biosystems, Foster City, CA, USA) in seven pairs of sisters of similar age (#15/16, 5/14, 6/11, 7/12, 8/13, 26/27, 33/34; see Table 1). Micro-fluidic real-time quantitative PCR cards were customized to include single-plex assays for all candidate genes obtained with DNA methylation, CNV, and GEX analyses. Genes reported by GWAS studies were also included among the candidates (7, 9, 24, 25). All samples were analyzed in duplicate, and included 94 candidate genes and the 18S and β-actin housekeeping genes. Analyses were performed using the ABI Prism 7900HT Sequence Detection System (SDS 2.2.2 software, Applied Biosystems, Foster City, CA, USA). PCR cycle conditions included 50°C for 2 min, 94.5°C for 10 min, and 40 cycles of 97°C for 30 s followed by 59.7°C for 1 min. The preliminary study of all 10 samples defined the maximum allowable cycle threshold (CT) that was set at 38 while outliers exceeding this threshold were excluded from the statistical analysis and no adjustment of *p*-value was performed. Internal controls for calculating expression levels of candidate genes were 18S and ACTB (beta-actin). The analysis has been performed with Data Assist version 3 statistical software (Applied Biosystems). The software exports data from real-time PCR and performs relative quantification analysis. The data assist analysis contains: *C*_t_ data, sample design, assay design, average of *C*_t_ values of replicates, Δ*C*_t_, normalized versus endogenous controls *C*_t_ values ± SD and fold change (RQ) files, which displays RQ min and RQ max for each sample. *p*-Value is calculated from Δ*C*_t_ files.

A heat map is used to visualize the data and illustrates, for all case/control sibling pairs, GEX in red/green color based on Δ*C*_t_ values using Pearson’s correlation. The neutral/middle expression was set as the median of all the Δ*C*_t_ values from all samples, the red indicated an increase with a Δ*C*_t_ value below the middle level and the green indicated a decrease with Δ*C*_t_ above the middle level_._

### Pathway analysis

Gene networks were generated through the use of Ingenuity Pathways Analysis software 8.0. Edition (Ingenuity Systems, http://www.ingenuity.com). Each gene identified was mapped to its corresponding gene object in the Ingenuity Pathways Knowledge Base and overlaid onto a global molecular network. The SDS 2.2.2 software (Applied Biosystems, Foster City, CA, USA) was used to determine changes in expression of a target in an experimental sample relative to the same target in a reference sample with the Student’s *t*-test and *p*-value <0.05 were considered statistically significant. We utilized Data Assist Software version 3 statistical software (Applied Biosystems) and Stata 8.0 for MacIntosh (Stata Corp, College Station, TX, USA) for statistical analyses.

## Results

### DNA methylation

DNA methylation comparison showed 60 differentially methylated regions (DMR) in affected compared to the non-affected twin (*p* < 0.05 for each of the three discordant twin pairs). These DMR corresponded to 51 genes on the X chromosome and 9 genes on autosomal chromosomes, listed in Table 2. For each DMR, the PBC proband was hypermethylated compared with the non-affected twin.

**Table 2:** **Differentially methylated genes in PBC-discordant MZ twins**.

Gene	Chr/base pair (bp)^a^	Description/function	Localization^b^
ABCD1	chrX:152989993–152991024	ATP-binding cassette, sub-family D (ALD), member 1	PM
ATP12A	chr13:25254828–25254890	ATPase, H+/K+ transporting, non-gastric, alpha polypeptide	PM
ATP5A1	chr18:43678161–43678731	ATP synthase, H+ transporting, mitochondrial F1 complex, alpha subunit 1, cardiac muscle	C
BCAP31	chrX:152989493–152990063	B cell receptor-associated protein 31	C
BGN	chrX:152760629–152761596	Biglycan	E
BRCC3	chrX:154299261–154299925	BRCA1/BRCA2-containing complex, subunit 3	N
CFP	chrX:47483418–47483642	Complement factor properdin	E
CHST7	chrX:46434647–46434858	Carbohydrate (*N*-acetylglucosamine 6-*O*) sulfotransferase 7	C
CTAG1A, CTAG1B	chrX:153813591–153814161	Cancer/testis antigen 1A, B	C
DDX41	chr5:176943911–176944481	DEAD (Asp–Glu–Ala–Asp) box polypeptide 41	N
FAM104B	chrX:55187570–55188140	Family with sequence similarity 104, member B	U
FGD1	chrX:54521696–54522266	FYVE, RhoGEF, and PH domain containing 1	C
FUNDC2	chrX:154255133–154255703	FUN14 domain containing 2	C
GAGE12B, 12I, 2A, 5, 7, 8	chrX:49315376–49315946	G antigen 1, 5, 7	U
GTPBP6	chrX:230686–231256	GTP-binding protein 6 (putative)	U
HCCS	chrX:11129525–11129638	Holocytochrome *c* synthase	C
HOXD4	chr2:177016716–177017157	Homeobox D4	N
IDH3G	chrX:153059742–153059944	Isocitrate dehydrogenase 3 (NAD+) gamma	C
IDS	chrX:148586616–148587185	Iduronate 2-sulfatase	C
IRAK1	chrX:153285317–153285887	Interleukin-1 receptor-associated kinase 1	PM
KBTBD6	chr13:41706829–41707399	Kelch repeat and BTB (POZ) domain containing 6	U
MAGEA3	chrX:151938154–151938356	Melanoma antigen family A, 3	U
MAGEA6	chrX:151867135–151867705	Melanoma antigen family A, 6	U
MAGEA9	chrX:148793401–148793568	Melanoma antigen family A, 9	U
MAGED4B	chrX:51928209–51929228	Melanoma antigen family D, 4B	U
MTCP1	chrX:154299410–154299612	Mature T cell proliferation 1	C
MTM1	chrX:149737348–149737918	Myotubularin 1	C
MTMR8	chrX:63614954–63615524	Myotubularin-related protein 8	U
NHS	chrX:17393481–17393959	Nance–Horan syndrome (congenital cataracts and dental anomalies)	N
ORC1L	chr1:52869831–52870401	Origin recognition complex, subunit 1	N
CDK16	chrX:47078470–47079428	Cyclin-dependent kinase 16	C
PDZD4	chrX:153095693–153096406	PDZ domain containing 4	C
PHF16	chrX:46772444–46773014	PHD finger protein 16	N
PRKX	chrX:3631431–3632001	Protein kinase, X-linked	C
PRPF38A	chr1:52869831–52870401	PRP38 pre-mRNA processing factor 38 (yeast) domain containing A	N
RIBC1	chrX:53449681–53450600	RIB43A domain with coiled-coils 1	U
RNF128	chrX:105970276–105970478	Ring finger protein 128	C
SCLY	chr2:238969783–238970252	Selenocysteine lyase	C
SHROOM4	chrX:50557007–50557209	Shroom family member 4	PM
SLC10A3	chrX:153718280–153718749	Solute carrier family 10 (sodium/bile acid cotransporter family), member 3	PM
SLC9A6	chrX:135067977–135068547	Solute carrier family 9 (sodium/hydrogen exchanger), member 6	PM
SLITRK2	chrX:144903417–144903908	SLIT and NTRK-like family, member 2	U
SLITRK4	chrX:142722571–142723141	SLIT and NTRK-like family, member 4	U
**SMARCA1**	chrX:128657308–128657936	SWI/SNF-related, matrix-associated, actin-dependent regulator of chromatin, sub-family A, member 1	N
SSR4	chrX:153060191–153060761	Signal sequence receptor, delta (translocon-associated protein delta)	C
TAF9B	chrX:77394695–77395265	TAF9B RNA polymerase II, TATA box-binding protein-associated factor, 31 kDa	N
TCEAL6	chrX:101397122–101397692	Transcription elongation factor A (SII)-like 6	U
TUSC3	chr8:15397909–15398479	Tumor suppressor candidate 3	PM
UBL4A	chrX:153714886–153715456	Ubiquitin-like 4°	U
VCX2, VCX3A	chrX:6451316–6452154	Variable charge, X-linked 2, X-linked 3A	U, N
YIPF6	chrX:67718891–67718965	Yip1 domain family, member 6	C
ZIC3	chrX:136649002–136649910	Zic family member 3 (odd-paired homolog, *Drosophila*)	N
ZNF182	chrX:47862911–47863428	Zinc finger protein 182	N

*Of note, all regions were hypermethylated in the PBC proband and only *SMARCA1* was differentially expressed in RT-PCR*.

*^a^Positions of each gene based on GRCh37/hg19*.

*^b^For each gene product the localization is specified as nuclear (N), cytoplasmic (C), plasma membrane (PM), extracellular (E), unknown (U)*.

### Copy number variations

Ten CNV were discordant between affected and the non-affected twin in one twin set. The healthy twin had four CNVs that were missing in the affected twin and six CNVs were present only in the affected twin. The CNVs were found in the following genes: *RYBP* ring 1, YY1 binding protein, *HERV-V2* envelope glycoprotein ENVV2, *POTEK P* ankirin domain family member K pseudogene, *THSD7A* thrombospondin type 1 domain containing 7A = *KIAA0960*, *GOLGA8A* golgin A8 family member A, *BPTF* bromodomain PHD finger transcription factor, and *C17orf58* open reading frame. Two additional CNV did not correspond to known genes.

### Microarray gene expression

Gene expression analysis using the genome-wide microarray showed two genes significantly down-regulated in PBC compared to the healthy sister in each of the eight discordant sister pairs. These genes were *IFIT1* (interferon-induced protein with tetratricopeptide repeats; chromosome 10q23.31) and *IFI44L* (interferon-induced protein 44-like; chromosome 1p31.1) and both are interferon-induced (28).

### RT-PCR analysis

**Figure 1:**
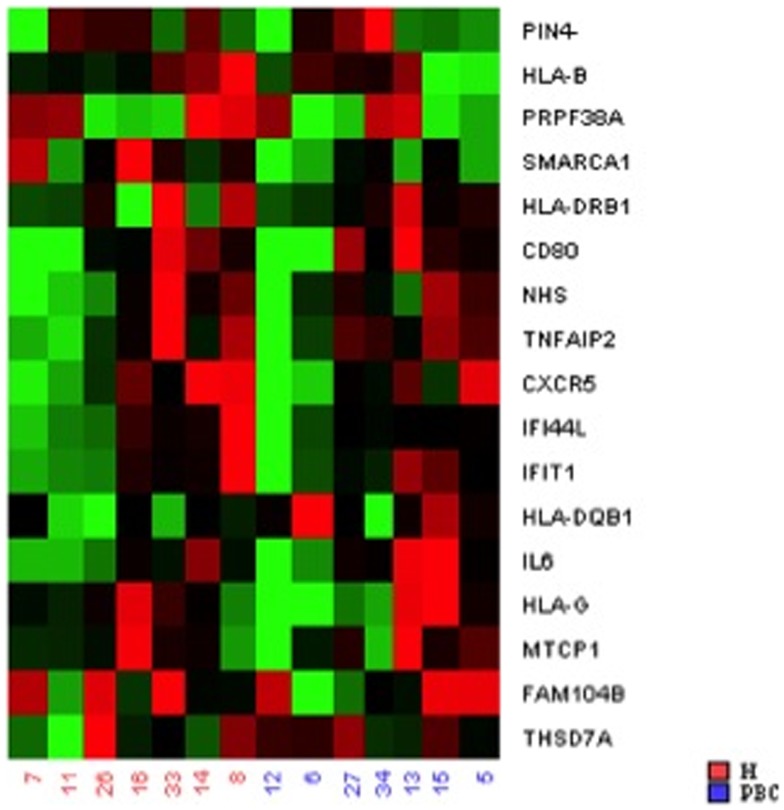
**Heat map showed, for all case/control sibling pairs, genes expression in red/green color based on Δ*C*_t_ values using Pearson’s correlation**. The red indicated an increased expression with a Δ*C*_t_ value below the middle level and the green indicated a decreased expression with Δ*C*_t_ value above the middle level.

**Figure 2:**
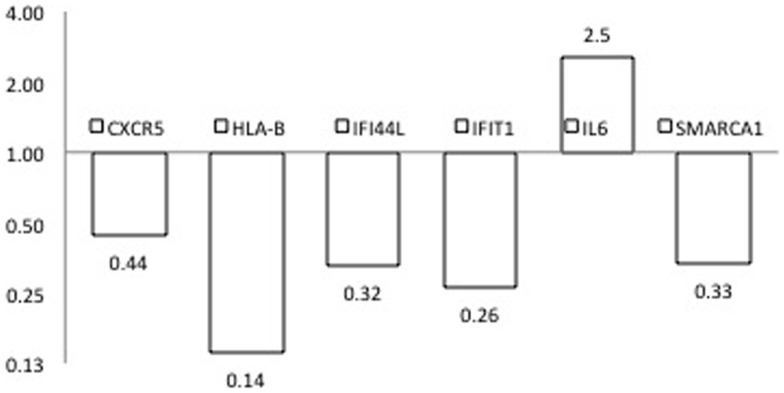
**Expression fold changes at RT-PCR of the six genes found differences in PBC versus healthy sibling pairs analysis subjects**.

To provide additional support for our initial findings, we used RT-PCR to evaluate expression of each of the candidates that emerged from the DNA methylation (60), CNV (10), and expression studies (2), as well as previously reported GWAS in seven pairs of discordant sisters of similar age (Table 1) (7–9, 12, 13, 24, 25). Our data assist analysis contained: *C*_t_ data, sample design, assay design, average of *C*_t_ values of replicates, Δ*C*_t_, normalized versus endogenous controls *C*_t_ values ± SD and fold change (RQ) files, which displays RQ min and RQ max for each sample. *p*-Value was calculated from Δ*C*_t_ files. Data assist v3.0 software was used with results exported from real-time PCR and for relative quantification analysis. Graphic result in heat map visualized analyzed data (Figure 1). Heat map showed, for all case/control sibling pairs, genes expression in red/green color based on Δ*C*_t_ values using Pearson’s correlation. The neutral/middle expression was set as the median of all the Δ*C*_t_ values from all samples, the red indicated an increase with a Δ*C*_t_ value below the middle level and the green indicated a decrease with Δ*C*_t_ value above the middle level. The heat map from all samples is represented in Figure 1. Among the entire set of candidate genes, we found five genes that were underexpressed in at least three of seven sibling pairs with FC < 0.5 (*CXCR5*, *HLA-B*, *IFI44L*, *IFIT1*, *SMARCA1*) and one overexpressed gene in at least three of seven pairs with an FC > 2 (*IL6*). Additional 11 genes showed a widely variable expression profile in each sibling pair (*CD80*, *FAM104B*, *HLA-DQB1*, *HLA-DRB1*, *HLA-G*, *MTCP1*, *NHS*, *PIN4*, *PRPF38A*, *THSD7A*, and *TNFAIP2*) (Table 3; Figure 2).

**Table 3:** **Genes showing consistent differences in DNA methylation, CNV, or expression^a^**.

Analysis	ID	Status^b^	Entrez gene name	Chr/base pair (bp)	Localization^c^
GWAS	CXCR5	Down-regulated in three sibling pairs 0.44	Chemokine (C–X–C motif) receptor 5	chr11: 118764101–118766980	PM
GWAS	HLA-B	Down-regulated in three sibling pairs 0.14	Major histocompatibility complex, class I, B	chr6: 31321649–31324989	PM
GEX	IFI44L	Down-regulated in four sibling pairs 0.32	Interferon-induced protein 44-like	chr1: 79086088–79111830	U
GEX	IFIT1	Down-regulated in three sibling pairs 0.26	Interferon-induced protein with tetratricopeptide repeats 1	chr10: 91152303–91166244	C
GWAS	IL6	Up-regulated in three sibling pairs 2.5	Interleukin 6 (interferon, beta 2)	chr7: 22766798–22771620	E
MeDIP	SMARCA1	Down-regulated in three sibling pairs 0.33	SWI/SNF-related, matrix-associated, actin-dependent regulator of chromatin, sub-family a, member 1	chrX: 128484989–128485617	N

*^a^List of genes evaluated with RT-PCR*.

*Each of the tested genes showed consistent differences in methylation (MeDIP), copy number variation (CNV), or gene expression (GEX) (in the current study) or were candidate genes from genome-wide association studies (GWAS) studies*.

*^b^Status: Log(RQ) is the logarithm of fold change = , which identifies the expression ratio: a positive Log(RQ) implies that the gene is up-regulated*.

*^c^For each gene product the localization is specified as nuclear (N), cytoplasmic (C), plasma membrane (PM), extracellular (E), unknown (U)*.

### Pathway analysis

Pathway analyses were performed using the 17 resulting genes from our study and demonstrated that the most representative functions and diseases were inflammatory, immunological, and connective tissue disorders. Furthermore the top canonical pathways involved were: T helper cell differentiation (*p* = 3.98E−19), dendritic cell maturation (*p* = 1.39E−13), altered T and B cell signaling in rheumatoid arthritis (*p* = 1.02E−12), type I diabetes mellitus signaling (*p* = 1.04E−11), and the crosstalk between dendritic cells and natural killer cells (*p* = 5.98E−11) (Table 4).

**Table 4:** **List of PBC-associated genes analyzed with ingenuity pathways analysis software 8.0 IPA**.

Genes	IPA findings
*IL6*	Up-regulation of human IL6 protein in serum is associated with human PBC
*IL4*	Up-regulation of human IL4 mRNA in liver is associated with human PBC
*IL17A*	Up-regulation of human IL-17 (IL17A) mRNA in liver is associated with human PBC
*IL13*	Up-regulation of human IL13 mRNA in liver is associated with human PBC
*IL12RB2*	Mutant human IL12RB2 gene (SNP substitution mutation (rs3790567) is associated with human PBC (*p*-value = 2.76E−11)
*IL12*	Mutant human IL12A gene [SNP substitution mutation, allelic variations: A/G (rs4679868)] is associated with human PBC
	Mutant human IL12A gene (SNP substitution mutation (rs574808) is associated with human PBC (*p*-value = 1.88E−13)
*HLA-DQB1*	Mutant human HLA-DQB1 gene (SNP substitution mutation (rs9275312) is associated with human PBC
	Mutant human HLA-DQB1 gene (SNP substitution mutation (rs2856683) is associated with human PBC (*p*-value = 1.78E−19)
	Mutant human HLA-DQB1 gene (SNP substitution mutation (rs7775228) is associated with human PBC
	Mutant human HLA-DQB1 gene (SNP substitution mutation (rs9275390) is associated with human PBC
	Mutant human HLA-DQB1 gene (SNP substitution mutation (rs9357152) is associated with human PBC
*HLA-DPB1*	Mutant human HLA-DPB1 gene (SNP substitution mutation (rs9277535) is associated with human PBC
	Mutant human HLA-DPB1 gene (SNP substitution mutation (rs2281389) is associated with human PBC
	Mutant human HLA-DPB1 gene (SNP substitution mutation (rs660895) is associated with human PBC
	Mutant human HLA-DPB1 gene (SNP substitution mutation (rs9277565) is associated with human PBC
*CTLA4*	Mutant human CTLA4 gene is associated with human PBC

*Of these, *IL6* was found differentially expressed by RT-PCR in discordant sisters*.

## Discussion

Primary biliary cirrhosis is considered a prototypic autoimmune disease because of the clinical homogeneity between patients and the relative consistency in natural history and pathology. Although relatively uncommon, several independent GWAS (7–13) have identified associations with transcription factors that further suggest a potential role for epigenetic shifts and thus our approach using this unique collection of DNA is a particularly important resource. We are aware of the numerous limitations of our study and that the observed changes in GEX may be stochastic rather than secondary to disease progression or involved in pathways involved in PBC pathogenesis, as suggested for other autoimmune diseases (29–32). The latter includes the possibility of portal hypertension and resulting leukopenia.

We identified 60 DMR and 10 CNV between discordant MZ twins with 14 (20%) also differently expressed between PBC cases and control sisters, thus being stronger candidates as PBC biomarkers or determinants. One of the strengths of our study is the confirmation of identified genes by quantitative PCR and that this approach was extended also to genes identified in recent GWAS allowing identification of six genes differently expressed in PBC mononuclear cells. First, these genes support a down-modulation of Th2-cytokines such as *IFIT1*, an interferon type I signature represented by *IFI44L*, in favor of a fibrogenic phenotype as represented by the *IL6* up-regulation (33). Regarding this last observation, we note the apparent discrepancy between DNA methylation and GEX of *IL6* but we recognize that methylation does not fully correlate with GEX, and the difference could be explained by different mechanisms such as allele-specific methylation (34, 35) (Table 4). Second, a single DMR-associated gene, i.e., hypermethylated *SMARCA1*, manifested a reduced GEX confirmed in our RT-PCR study of sibling pairs. *SMARCA1* is a transcription regulator that modulates the chromatin structure and is involved in apoptosis, DNA damage, and differentiation. Moreover, the gene encodes for a member of the *SWI/SNF* family of proteins, which are master regulators of GEX, regulating expression among others *FOS*, *CSF-1*, *CRYAB*, *MIM-1*, *p21* (also known as *CDKN1A*), *HSP70*, *VIM*, and *CCNA2*; *SWI/SNF* has also been reported to modulate alternative splicing (36). Third, 5/7 sibling pairs had consistent dysregulation of *CXCR5* being down-regulated in PBC lymphocytes, which may reflect a compartmentalization of *CXCR5*+ cells within the liver or may reflect the chronic activation of B cells, as reported in rheumatoid arthritis (37). In fact, the chemokine receptor *CXCR5* is expressed by B and T cells and controls their migration within lymph nodes while its ligand *BCA-1/CXCL13* is present in lymph nodes and spleen and also in the liver. A downregulation of *CXRC5* is correlated with an increased production of *IL-2*, which may cause the production of immunoglobulins by B cells; *IL-2* is normally produced by T cells during an immune response. *IL-2* is also necessary during T cell differentiation in Treg, which are involved in self antigens recognition, which could result in autoimmunity (38). Of note, following B cell activation and differentiation into plasma cells and memory cells, *CXCR5* becomes down-regulated while the same effect is induced *in vitro* following anti-CD40 stimulation (39) and *CD40L* methylation appears to be altered in PBC (40). Fourth, *HLA-B* is also down-regulated in PBC, similar to several types of cancer (41–43).

The majority of the identified genes map on the X chromosome, in agreement with the female predominance of the disease, and are involved in many cellular pathways. Our group in a previous work assessed the expression of 125 genes with variable X inactivation status and found that two genes (*CLIC2* and *PIN4*) were consistently down-regulated in PBC affected twin of discordant pairs (17). Three genes are differentially methylated in lymphocytes of patients with PBC and systemic sclerosis (32) and may thus be representative of general autoimmunity or fibrosis development; these genes include *MTM1* hypermethylated in PBC and in systemic sclerosis while *SSR4* and *IGH3G* are hypomethylated in both diseases. Of note, a recent study reported the up-regulation of the X-linked costimulatory molecule *CD40L* (40) but our data failed to confirm such hypomethylation in our cohort. The CNV differences observed in our MZ twin set warrant some further observations as the *de novo* post-twinning CNV frequency was estimated to be as high as 5% on a per-individual basis or 10% per twinning event (21). While the impact of CNV on GEX can vary (44), it would be of great interest to obtain parental information to determine the origin and timing of CNV in the offspring. On the other hand, there are several limitations to our data. PBC is relatively uncommon and our DNA collection reflects a several-year worldwide search; it is nonetheless a limited dataset. In addition, there is only limited information available using PBMC. PBC is an organ-specific disease affecting small intrahepatic bile ducts and thus studies of the portal infiltrating lymphocytes will provide a more valuable resource as would a detailed and well-defined lymphoid cell populations. These comments notwithstanding, the data obtained are intriguing and consistent with our thesis that one explanation for discordant MZ twins is DNA changes on the critical genomic element involved in disease susceptibility and these observations should be recapitulated also in unrelated pairs of patients and controls. With the increased interest in the balance between genetic susceptibility, it becomes critical for research groups to combine resources and improve access to clinical material and data that permits more extensive studies and the potential for more powerful statistical analysis and interpretation.

## Conflict of Interest Statement

The authors declare that the research was conducted in the absence of any commercial or financial relationships that could be construed as a potential conflict of interest.
