# Corrigendum: Genome-Wide Analysis of DNA Methylation, Copy Number Variation, and Gene Expression in Monozygotic Twins Discordant for Primary Biliary Cirrhosis

**DOI:** 10.3389/fimmu.2014.00371

**Published:** 2014-08-14

**Authors:** Carlo Selmi, Francesca Cavaciocchi, Ana Lleo, Cristina Cheroni, Raffaele De Francesco, Simone A. Lombardi, Maria De Santis, Francesca Meda, Maria Gabriella Raimondo, Chiara Crotti, Marco Folci, Luca Zammataro, Marlyn J. Mayo, Nancy Bach, Shinji Shimoda, Stuart C. Gordon, Monica Miozzo, Pietro Invernizzi, Mauro Podda, Rossana Scavelli, Michelle R. Martin, Michael F. Seldin, Janine M. LaSalle, M. Eric Gershwin

**Affiliations:** ^1^Division of Rheumatology and Clinical Immunology, Humanitas Clinical and Research Center, Milan, Italy; ^2^Division of Rheumatology, Allergy, and Clinical Immunology, University of California at Davis, Davis, CA, USA; ^3^BIOMETRA Department, University of Milan, Milan, Italy; ^4^Liver Unit and Center for Autoimmune Liver Diseases, Humanitas Clinical and Research Center, Milan, Italy; ^5^National Institute of Molecular Genetics (INGM), Milan, Italy; ^6^University of Texas Southwestern, Dallas, TX, USA; ^7^Mt. Sinai University, New York, NY, USA; ^8^Clinical Research Center, National Nagasaki Medical Center, Nagasaki, Japan; ^9^Henry Ford Hospital, Detroit, MI, USA; ^10^Department of Pathophysiology and Transplantation, University of Milan, Milan, Italy; ^11^Division of Pathology, Fondazione IRCCS Cà Granda Ospedale Maggiore Policlinico, Milan, Italy; ^12^Genome Center and M.I.N.D. Institute, University of California at Davis, Davis, CA, USA; ^13^Department of Biochemistry and Molecular Medicine, University of California at Davis, Davis, CA, USA; ^14^Department of Internal Medicine, University of California at Davis, Davis, CA, USA

**Keywords:** autoimmune cholangitis, epigenetics, environment, DNA methylation, PBC

Author list for the article “Genome-wide analysis of DNA methylation, copy number variation, and gene expression in monozygotic twins discordant for primary biliary cirrhosis” should be as follows:

Carlo Selmi^1,2^*, Francesca Cavaciocchi^1,3^, Ana Lleo^4^, Cristina Cheroni^5^, Raffaele De Francesco^5^, Simone A. Lombardi^1^, Maria De Santis^1,3^, Francesca Meda^1^, Maria Gabriella Raimondo^1^, Chiara Crotti^1^, Marco Folci^1^, Luca Zammataro^1^, Marlyn J. Mayo^6^, Nancy Bach^7^, Shinji Shimoda^8^, Stuart C. Gordon^9^, Monica Miozzo^10,11^, Pietro Invernizzi^4^, Mauro Podda^1^, Rossana Scavelli^5^, Michelle R. Martin^12^, Michael F. Seldin^13,14^, Janine M. LaSalle^12^, and M. Eric Gershwin^2^

^1^Division of Rheumatology and Clinical Immunology, Humanitas Clinical and Research Center, Milan, Italy

^2^Division of Rheumatology, Allergy, and Clinical Immunology, University of California at Davis, Davis, CA, USA

^3^BIOMETRA Department, University of Milan, Milan, Italy

^4^Liver Unit and Center for Autoimmune Liver Diseases, Humanitas Clinical and Research Center, Milan, Italy

^5^National Institute of Molecular Genetics (INGM), Milan, Italy

^6^University of Texas Southwestern, Dallas, TX, USA

^7^Mt. Sinai University, New York, NY, USA

^8^Clinical Research Center, National Nagasaki Medical Center, Nagasaki, Japan

^9^Henry Ford Hospital, Detroit, MI, USA

^10^Department of Pathophysiology and Transplantation, University of Milan, Milan, Italy

^11^Division of Pathology, Fondazione IRCCS Cà Granda Ospedale Maggiore Policlinico, Milan, Italy

^12^Genome Center and M.I.N.D. Institute, University of California at Davis, Davis, CA, USA

^13^Department of Biochemistry and Molecular Medicine, University of California at Davis, Davis, CA, USA

^14^Department of Internal Medicine, University of California at Davis, Davis, CA, USA

The original article has been updated.

## Conflict of Interest Statement

The authors declare that the research was conducted in the absence of any commercial or financial relationships that could be construed as a potential conflict of interest.

